# Clinical and Endoscopic Differences Between Patients With Barrett’s Esophagus With and Without Dysplasia/Adenocarcinoma

**DOI:** 10.7759/cureus.46323

**Published:** 2023-10-01

**Authors:** Francisco Valdovinos-Andraca, Isaac Bartnicki-Navarrete, Ambrosio R Bernal-Mendez, Rafael Rafael Barreto-Zuñiga, Adriana F Romano-Munive, Armando Gamboa-Domínguez, Javier Elizondo-Rivera, Daniel Briseño-García, Felix I Tellez-Ávila

**Affiliations:** 1 Endoscopy, National Institute of Medical Sciences and Nutrition Salvador Zubiran, Mexico City, MEX; 2 Pathology, National Institute of Medical Sciences and Nutrition Salvador Zubiran, Mexico City, MEX; 3 Gastroenterology and Hepatology, University of Arkansas for Medical Sciences, Little Rock, USA

**Keywords:** barrett's esophagus, mexico, risk factors, esophageal adenocarcinoma, dysplasia

## Abstract

Introduction: Barrett's esophagus (BE) is the main precursor of esophageal adenocarcinoma (EAC). This study aimed to identify the risk factors associated with BE progression to dysplasia or EAC in a Latin population.

Methods: The study is a retrospective analysis of a single-center cohort of patients with BE, evaluated from 2002 to 2012.

Results: We identified 420 patients with BE; 281 (66.9%) of them were men with a mean age of 57.2 ± 15.3 years. Among all BE patients evaluated, 81 (19.3%) had progression to some degree of dysplasia/EAC. The mean follow-up was 5.6 years. Multivariate analysis showed that age (OR = 1.03), cigarette smoking (OR = 3.05), long-segment BE (OR = 4.81), and a visible lesion on BE (OR = 6.94) were associated with progression to dysplasia/EAC.

Conclusion: In Latin patients with BE, age, cigarette smoking, long-segment BE, and the presence of lesions were associated with the presence of dysplasia/EAC.

## Introduction

Barrett's esophagus (BE) is a metaplastic change in the distal esophagus involving the replacement of the normal squamous epithelium by a specialized intestinal columnar epithelium. It is the main known precursor lesion of esophageal adenocarcinoma (EAC) [1,2]. In the general population, its prevalence is estimated at 1-2%. It is found in 10-15% of all patients with gastroesophageal reflux symptoms of several years of evolution [3]. In Mexico, BE studies have reported a prevalence of 0.26%, 1.8%, and 9.6% [4-6]. Compared with the general probability, patients with BE have a 50 to 55 times higher risk of developing EAC [7]. In addition, the annual risk of progression of BE to EAC in patients without dysplasia is 0.33% [8]. However, in BE patients with low- and high-grade dysplasia, the annual rates of progression to EAC could be as high as 1% and 6%, respectively [9]. In recent decades, the incidence of EAC has been on the increase, while the five-year survival prognosis is less than 15% [10,11].

Since BE is the known precursor of EAC, it is pertinent that once diagnosed, an initial evaluation be performed and endoscopic follow-up instituted, both with the aim of early detection of cancer or dysplasia, and hence subsequent curative treatment. Nevertheless, consensus is lacking on the follow-up intervals between the different medical societies [12-15]. In addition, it should be borne in mind that there is no conclusive evidence of the benefits and the impacts on mortality of these actions [16]. Attempts have been made to establish the risk factors for progression from BE to EAC. In a multicenter longitudinal study of the Caucasian and Anglo-Saxon populations, the authors identified male gender, smoking, BE length, and initial presence of low-grade dysplasia as risk factors for progression [17,18]. A meta-analysis found similar results but included “age at diagnosis” as a risk factor for progression [18]. In our population, these risk factors are unknown. Detecting factors associated with the presence or development of EAC in BE cases could impact the different screening and prevention strategies in our population. On this basis, the objective of the present study was to identify differences between patients with and without dysplasia/EAC in patients with BE in a Latin population.

## Materials and methods

It is a comparative retrospective study. Patients with a histological diagnosis of BE from January 2002 to December 2012 were included. BE data were collected from the databases of the Gastrointestinal Endoscopy and Pathology Departments of the National Institute of Medical Sciences and Nutrition, Salvador Zubirán (INCMNSZ) in Mexico City. Affiliated physicians and residents were responsible for performing the endoscopic procedures. Biopsies were obtained using white-light, high-definition Olympus GIF 160® and 180® model endoscopes (Olympus Corporation, Tokyo, Japan), with the application of vital chromoendoscopy or narrow-band imaging (NBI) at the discretion of the endoscopist, based on the Seattle protocol and with biopsies directed at observed lesions (nodule, stricture, and ulcer) within the BE. All procedures were done under moderate sedation or monitored anesthesia care. The biopsies were processed in accordance with the standard protocol of the INCMNSZ Pathology Department and reviewed by physicians assigned to this department.

In all the patients, data on age, sex, body mass index (BMI), the presence or absence of tobacco use, alcohol, proton pump inhibitors (PPIs), non-steroidal anti-inflammatory drugs (NSAIDs), presence of hiatal hernia, Nissen fundoplication, and symptoms of gastroesophageal reflux at BE diagnosis (based on patient history documenting heartburn or regurgitation) were recorded. In the endoscopic studies, the length of the BE was documented and divided into short-segment BE (less than 3 cm) and long-segment BE (greater than or equal to 3 cm). The presence of lesions, defined as a nodule, stricture, and ulcer, within the BE was recorded. Our hospital follow-up recommendation for patients with BE is based on clinical management guidelines, which depend on the type of histopathological results obtained [12-14].

The samples were divided, considering the pathology report at study entry, into two groups: BE without dysplasia (only intestinal metaplasia) and BE with dysplasia (low-grade dysplasia, high-grade dysplasia, and the presence of EAC). Subsequently, the variables, previously mentioned, were compared to establish significant differences and to identify variables associated with the presence of dysplasia/EAC.

In all patients, the follow-up time was documented and changes or no changes of BE without dysplasia to BE with dysplasia/EAC and vice versa were recorded. In addition, patients' treatment and type or no treatment were registered.

Statistical analysis

Descriptive statistics with measures of central tendency and dispersion for a population with a parametric or non-parametric distribution that is appropriate for the variable were used. To evaluate differences between the groups, we used chi-square or Fisher's exact test for categorical variables and Student's t-test for continuous variables. Variables with a p-value < 0.2 were included in the multivariate analysis. A p-value <0.05 was considered significant. SPSS v20 statistical program (IBM Corp., Armonk, NY) was used.

Ethical considerations

This study was performed with strict observance of the bioethical precepts of human research and Helsinki principles. The study consisted of a retrospective review of data from INCMNSZ files and collected at the moment of review. Due to the retrospective nature and review of the data in the file, the consent of each of the patients involved was not necessary. However, care was taken at all times to guard the anonymity of the patients. The protocol was approved by the INCMNSZ Ethics and Research Committee with registration number END-3085-20-21-1.

## Results

A total of 43,639 upper digestive tract endoscopies were performed in the period evaluated and 420 patients were diagnosed with BE (9.6 patients with BE for every 1000 studies performed); 66.9% were men with mean age ± SD of 57.2 ± 15.3 years, 223 (53%) of the patients presented long-segment BE and 197 (47%) with short-segment BE, 339 (80.7%) were found without dysplasia, while 81 (19.3%) had some degree of dysplasia or cancer. In Table 1, the clinical and endoscopic characteristics of included patients are shown.

**Table 1:** Clinical and endoscopic characteristics of BE patients without dysplasia and BE patients with dysplasia/EAC. * Ulcer, estenosis, or nodule. BE: Barrett's esophagus; EAC: esophageal adenocarcinoma; BMI: body mass index; GERD: gastroesophageal reflux disease; PPI: proton pump inhibitor; NSAIDs: non-steroidal anti-inflammatory drugs. P-value < 0.05 was considered significant.

Characteristics	BE without dysplasia (n = 339), n (%)	BE with dysplasia/EAC (n = 81), n (%)	P
Gender, male	223 (65.7)	58 (71.6)	0.5
Age, years (mean ± SD)	56.3± 15.4	62.5 ± 13.3	0.002
BMI kg/m2 (mean ± SD)	26± 5	26.3 ± 3.9	0.66
Smoking	177 (52)	62 (76.5)	0.001
Alcohol	126 (37.2)	40 (49.4)	0.011
GERD symptoms	254 (74.9)	64 (79)	0.06
PPI	183 (53.9)	52 (64.2)	0.14
NSAIDs	37 (11)	13 (16)	0.29
Nissen surgery	55 (16.2)	8 (9.9)	0.052
Hiatal hernia	219 (64.6)	52 (64)	0.75
Lesion*	30 (8.8)	25 (30.9)	0.001
BE large	163 (48)	62 (76.5)	0.001

Diagnosis and follow-up

According to the pathology report at study entry, the patients were divided into those without dysplasia (n = 339) and those with dysplasia/EAC (n = 81), including those with low-grade dysplasia and high-grade dysplasia/EAC. Twenty-one patients were diagnosed with high-grade dysplasia at study entry and in eight cases, high-grade dysplasia coexisted with EAC. Thirteen patients were diagnosed with EAC only, without dysplasia at biopsies.

In the follow-up of the 339 patients without dysplasia at study entry, 25 (7.4%) progressed to low-grade dysplasia and five to high-grade dysplasia (1.5%). For the 47 patients with low-grade dysplasia at baseline, 15 (31.9%) remained unchanged during follow-up, 29 (61.7%) subsequently presented BE without dysplasia, and four (8.5%) progressed to EAC. In those with high-grade dysplasia at study entry, one progressed to cancer, four remained unchanged (three received endoscopic treatment), three were reported with low-grade dysplasia after mucosectomy, and four underwent esophagectomy. The mean follow-up time was 5.6 years.

Univariate and multivariate analysis

In Table 2, univariate and multivariate analyses are shown. Age, tobacco use, BE lesions, and segment length ≥ 3 cm (long-segment BE) were associated with the presence of dysplasia/EAC.

**Table 2:** Univariate and multivariate analysis of the factors associated with the presence of dysplasia/EAC in patients with BE. BE: Barrett's esophagus; EAC: esophageal adenocarcinoma; OR: odds ratio; CI: confidence interval; BMI: body mass index; GERD: gastroesophageal reflux disease; PPI: proton pump inhibitor; NSAIDs: non-steroidal anti-inflammatory drugs. Variables with a p-value < 0.2 were included in the multivariate analysis. P-value < 0.05 was considered significant.

Characteristics	OR	CI	P
Univariate			
Age	1.02	1.004 – 1.039	0.014
Gender	0.76	0.448 – 1.298	0.32
Reflux	1.47	0.808 – 2.670	0.21
Smoking	2.99	1.712 – 5.210	<0.001
Alcohol	2.12	1.298 – 3.460	0.003
PPI	1.66	1.008- 2.747	0.047
NSAIDs	1.56	0.787 – 3.094	0.203
Lesion	6.36	3.507 – 11.53	<0.001
Hiatal hernia	1.11	0.665 – 1.853	0.69
Nissen	0.58	0.264 – 1.269	0.17
Length	4.77	2.649 – 8.573	<0.001
Multivariate			
Age	1.03	1.012 – 1.053	0.02
Smoking	3.05	1.65 – 5.66	<0.001
Lesion	6.94	3.56 – 13.45	<0.001
Length	4.81	2.52 – 9.12	<0.001

## Discussion

The results of the present study depict an association between age >60 years, tobacco use, the presence of lesions and long-segment BE, and the presence of dysplasia/EAC in biopsies from Mexican patients with BE.

Most of the patients (80.7%) in our study population did not present dysplasia. Of those with dysplasia (19.3%), low-grade dysplasia (11.2%) predominated in relation to high-grade dysplasia (5%). The evolution over time presented differences according to the type of dysplasia. It was observed that in the 339 patients without dysplasia at study entry, only 25 (7.4%) progressed to low-grade dysplasia and five to high-grade dysplasia (1.5%). Previous studies that monitored BE patients without dysplasia reported that the probability of dying from a cause unrelated to EAC is 10 times higher [8]. That is, BE patients without dysplasia have a lower risk of progressing to EAC (0.1% to 0.5% per patient-year) when compared with those with low- and high-grade dysplasia [19]. Although the increased risk of high-grade dysplasia progression to EAC is well established, there are studies where the rate of progression was reported to vary from 16% in a seven-year period to 50% in a five-year period [10,20]. A more recent meta-analysis in patients with BE estimated an annual incidence of EAC in patients with high-grade dysplasia of 6% [21].

An interesting group in the present study is patients with low-grade dysplasia; 15 (31.9%) of the 47 patients with this condition remained unchanged while 29 (61.7%) presented BE without dysplasia and four (8.5%) progressed to EAC at the end of their follow-up. A possible explanation for these situations may be sampling error in obtaining the endoscopic biopsies or inter-observer variability in the interpretation of the biopsies and/or the regularity in the analysis by two expert pathologists, situations that have been documented in other works [22,23]. This highlights the importance of adequately confirming both low-grade and high-grade dysplasia.

There are several reports where the degree of abdominal fat content has a greater impact on BE than BMI per se [24,25]. In our study, it was not possible to obtain the percentage of abdominal fat in our patients, and weight and BMI were not found to be associated with dysplasia/cancer in the patients evaluated.

Currently, surveillance intervals for BE > 3 cm are three years while shorter segments (<3 cm) could surveyed every five years. It is important to notice that endoscopic surveillance can conclude when the patient is not a good candidate for endoscopic eradication therapy. Our hospital followed international recommendations for patients with BE [26].

Management of BE with low-grade dysplasia includes endoscopic surveillance or endoscopic eradication therapy while management of BE with high-grade dysplasia includes endoscopic eradication therapy. In this study, four patients with high-grade dysplasia were managed with esophagectomy [26].

The present study has its limitations and strengths. Within the limitations, we can highlight the retrospective design and that some variables were categorized as binary variables (smoking, alcohol, use of PPIs, or NSAIDs), which could cause data loss. The BE groups with and without dysplasia are different risk populations, so they have different follow-ups. Another limitation is that the follow-up time is too short for the appropriate establishment of the progression of each group. Apart, the expert vs. resident variable in the detection of BE was not compared. Despite these limitations, this work is the largest series reported so far in our country, and where factors associated with the presence of dysplasia/EAC in patients with BE are evaluated.

## Conclusions

In conclusion, the results of the present study found an association between age, cigarette smoking, long-segment BE, and the presence of lesions in BE and progression of BE to dysplasia (30 of 339 patients, 8.8%) or dysplasia to EAC (five of 59 patients, 8.4%) in a Latin population during a mean follow-up of 5.6 years.
